# Metabolomic Profile of Weight Gain of People Living with HIV Treated with Integrase Strand Transfer Inhibitor Regimens Reveals Dysregulated Lipid Metabolism and Mitochondrial Dysfunction

**DOI:** 10.3390/metabo15110695

**Published:** 2025-10-25

**Authors:** Ana Miriam Ascencio-Anastacio, Violeta Larios-Serrato, José Antonio Mata-Marín, Mara Rodríguez Evaristo, Mireya Núñez-Armendáriz, Ana Luz Cano-Díaz, Alberto Chaparro-Sánchez, Gloria Elizabeth Salinas-Velázquez, Angélica Maldonado-Rodríguez, Javier Torres, María Martha García-Flores, Zuriel Eduardo Martínez-Valencia, Beatriz Irene Arroyo-Sánchez, Viridiana Olin-Sandoval, Fernando Minauro, Jesus Enrique Gaytán-Martínez, Ericka Nelly Pompa-Mera

**Affiliations:** 1 Infectious Diseases Department, Hospital de Infectología “Dr. Daniel Méndez Hernández”, Centro Médico Nacional “La Raza”, Instituto Mexicano del Seguro Social, Mexico City 02990, Mexico; anamiriam_1@comunidad.unam.mx (A.M.A.-A.); jamatamarin@gmail.com (J.A.M.-M.); mararodrigueze@gmail.com (M.R.E.); mire7invierno@gmail.com (M.N.-A.); ana.knodiaz@gmail.com (A.L.C.-D.); alberto.chaparro@imss.gob.mx (A.C.-S.); jesus.gaytan@imss.gob.mx (J.E.G.-M.); 2 Laboratorio de Biotecnología y Bioinformática Genómica, Escuela Nacional de Ciencias Biológicas (ENCB), Instituto Politécnico Nacional (IPN), Lázaro Cárdenas Professional Unit, Mexico City 11340, Mexico; vlarios@ipn.mx; 3 Radiology Department, Hospital de Infectología “Dr. Daniel Méndez Hernández”, Centro Médico Nacional “La Raza”, Instituto Mexicano del Seguro Social, Mexico City 02990, Mexico; gloriamed@gmail.com; 4 Unidad de Investigación Médica en Enfermedades Infecciosas, UMAE Pediatría, Centro Médico Nacional S.XXI, Instituto Mexicano del Seguro Social, Mexico City 06720, Mexico; mangimr1920@gmail.com (A.M.-R.); uimeip@gmail.com (J.T.); 5 Unidad de Investigación Médica en Inmunología, UMAE Pediatría, Centro Médico Nacional S.XXI, Instituto Mexicano del Seguro Social, Mexico City 06720, Mexico; marthamaria5234@gmail.com; 6 Department of Biotechnology and Bioengineering, Centro de Investigación y de Estudios Avanzados del Instituto Politécnico Nacional, Mexico City 07360, Mexico; eduardo.martinez@cinvestav.mx (Z.E.M.-V.); bety.arroyos@cinvestav.mx (B.I.A.-S.); viridiana.olin@cinvestav.mx (V.O.-S.); 7 Unidad de Investigación Médica en Genética Humana, UMAE Pediatría, Centro Médico Nacional S.XXI, Instituto Mexicano del Seguro Social, Mexico City 06720, Mexico; misaf73@gmail.com; 8 Research Unit, Hospital de Infectología “Dr. Daniel Méndez Hernández”, Centro Médico Nacional “La Raza”, Instituto Mexicano del Seguro Social, Mexico City 02990, Mexico

**Keywords:** plasma metabolome, HOMA-IR, acylcarnitines, amino acid metabolism, docosahexaenoic acid, circulating amino acid derivatives, HIV viral load, antiretroviral therapy

## Abstract

**Background/Objectives**: Excessive weight gain is a growing concern among people living with HIV (PWH) receiving integrase strand transfer inhibitor (INSTI)-based regimens as first-line antiretroviral therapy (ART), as it may contribute to multimorbidity. The mechanisms driving weight gain in INSTI users are not fully understood but are thought to be multifactorial. This study examines the plasma metabolome associated with weight gain in PWH on INSTI-based regimens. **Methods**: We conducted a nested case–control study within the randomized clinical trial MICTLAN (NCT06629480). Sixty-six participants were randomized to receive INSTI-based regimens, either bictegravir/tenofovir alafenamide/emtricitabine (BIC/TAF/FTC) or dolutegravir/abacavir/lamivudine (DTG/ABC/3TC), and followed for 18 months. Weight gain >10% relative to baseline was considered a primary endpoint and used as a criterium to categorize cases (n = 28) and controls (n = 38). Anthropometric and clinical measurements, plasma insulin, and metabolomic profiles were assessed at baseline and 18 months post-ART. Plasma untargeted metabolomics was performed using liquid chromatography–mass spectrometry (LC-MS/MS) to identify metabolomic changes linked to weight gain. Bioinformatic tools, including Partial Least Squares Discriminant Analysis (PLS-DA), volcano plots, and KEGG pathway enrichment analysis, were used to analyze plasma metabolomes and identify significant differential metabolites. **Results**: Weight gain at 18 months in PWH on INSTI-based ART was associated with insulin resistance, as measured by HOMA-IR (OR 3.23; 95% CI 1.14–9.10; *p* = 0.023), and visceral adipose tissue thickness > 4 cm (OR 4.50; 95% CI 1.60–13.03; 9.10; *p* = 0.004), and hypertriglyceridemia (OR 3.9; 95% CI 1.38–10.94; *p* = 0.008). Baseline HIV RNA viral load >50,000 copies/mL (OR 8.05; 95% CI 2.65–24.43; *p* = 0.0002) was identified as a baseline predictor of weight gain (aOR 6.58 (1.83–23.58); *p* = 0.004). In addition, accumulation of circulating medium-chain acylcarnitines, indicative of mitochondrial dysfunction, and insulin resistance were linked to weight gain in PWH on INSTI-based regimens after 18 months of therapy. **Conclusions**: This metabolomic study identified metabolites reflecting mitochondrial dysfunction, dysregulated lipid metabolism, and altered amino acid metabolism as key mechanisms underlying insulin resistance and weight gain in PWH on INSTI-based ART.

## 1. Introduction

Initiation of antiretroviral therapy (ART) in people living with HIV (PWH) promotes beneficial weight gain, resolving the debilitating catabolic state and restoring body fat and protein stores as part of immune reconstitution and the “return to health” condition [1]. However, the introduction of second-generation integrase strand transfer inhibitors (INSTIs) as first-line ART has led to excessive weight gain, contributing to increased inflammation and cardiovascular disease risk [2]. PWH with overweight or obesity exhibits a higher prevalence of multimorbidity, including type 2 diabetes mellitus (T2DM) [3]. Numerous pivotal studies have investigated weight changes over time and associated risk factors [3,4,5,6]. Overweight and obesity in PWH on INSTI-based ART are primarily characterized by central fat accumulation, with visceral adipose tissue (VAT) deposition in intra-abdominal, pericardial, liver, and skeletal muscle ectopic fat [7,8]. The pathophysiological mechanisms underlying these effects of INSTIs, such as dolutegravir (DTG), raltegravir (RAL), and bictegravir (BIC), have been explored. Studies in macaque models exposed to DTG and adipose tissue from PWH undergoing bariatric surgery have shown hyperplasia, hypertrophy, and hypoxia in adipocytes, along with increased fibrosis, oxidative stress, mitochondrial dysfunction, and insulin resistance in VAT and subcutaneous adipose tissue (SAT) [9,10]. Metabolomic approaches can identify alterations in tissue and circulating metabolites following ART exposure. A recent multiomic cross-sectional study of SAT and VAT biopsies from PWH with cardiometabolic disease risk revealed that, despite long-term virological suppression, metabolic reprogramming is associated with metabolites such as α-aminoadipate, butyryl carnitine, tyrosine, diacylglycerol, and acylcarnitines [11]. Despite its clinical significance, the mechanisms driving weight gain in PWH on INSTI-based ART remain poorly understood. Considering that the prevalence of overweight and obesity in people living with HIV could increase globally, with the use of INSTIs, the application of metabolomics offers a comprehensive, untargeted approach to identify distinctive metabolic signatures and identify the risk of this comorbidity, as well as guide therapeutic interventions.

This study aimed to identify metabolomic profiles and pathways associated with weight gain in PWH following 18 months of INSTI-based ART, to elucidate potential mechanistic insights. The results showed higher accumulation of circulating medium-chain acylcarnitines in PWH that experienced excessive weight gain after starting INSTI-based ART; which constitutes a metabolomic signature indicative of mitochondrial dysfunction..

## 2. Materials and Methods

### 2.1. Study Design and Subjects

We conducted a longitudinal pilot study with a nested case–control design within the MICTLAN clinical trial (ClinicalTrials.gov NCT06629480) [8,12]. The justification for conducting this pilot study is because this type of design allows for the assessment of treatment safety (in terms of excessive weight gain) and estimation of treatment effect and its variance (metabolomics) [13]. Given the nature of the pilot study, bias control was performed through the random assignment of participants to groups to ensure that the groups are comparable and minimize selection bias; use of standardized and calibrated methods for laboratory data collection; and use of multivariate models to control confounding variables and estimate the effect of the exposure of interest. This study adhered to the principles of the Declaration of Helsinki and was approved by the Investigation and Ethics Committee of IMSS (R-2023-785-032 and R-2021-3502-084). Participants were recruited from the Infectious Diseases Hospital “Daniel Méndez Hernández” at the National Medical Center “La Raza,” IMSS, Mexico City, between 2021 and 2024.

Sixty-six participants were randomized (1:1) to receive either bictegravir/tenofovir alafenamide/emtricitabine (BIC/TAF/FTC) or dolutegravir/abacavir/lamivudine (DTG/ABC/3TC). Treatment allocation was determined using a digital randomization system (MEDSHARING). A recruitment flowchart is presented in Figure S1.

Written informed consent was obtained from all participants prior to randomization and blood sampling. Enrolled patients were informed about the study’s nature and managed in accordance with our institution’s ethical guidelines.

Inclusion criteria were Mexican Mestizos (at least three generations born in Mexico, as a result of admixture of three founder populations: Amerindian, Spanish, and a smaller proportion of an African population [14]), with confirmed HIV diagnosis, age >18 years, and being ART-naïve. Exclusion criteria included prior ART exposure, antimicrobial therapy within the past 2 weeks, opportunistic infections, AIDS-defining conditions, neoplasia, use of medications associated with weight changes, ongoing treatment for tuberculosis or cancer, endocrinopathies, type 2 diabetes mellitus (T2DM), metabolic syndrome (MetS) per ATP III or IDF criteria, and coinfection with HBV, HCV, or HEV during follow-up.

Participants were followed for 18 months after initiating ART. As a primary endpoint, we considered weight gain > 10% relative to baseline to categorize participants into two groups: cases (n = 28), defined as those with weight gain > 10%, and controls (n = 38), defined as those with weight gain <10%.

### 2.2. Body Composition Measurements

Body composition, including weight, fat mass, muscle mass, bone mass, and body water, was evaluated at baseline (prior to initiating INSTI-based ART) and after 18 months of medical follow-up (post-INSTI-ART) using a next-generation bioimpedance device (TANITA FitScan BC-545F Segmental Body Composition Monitor (Tanita Corporation, Tokyo, Japan)). Adiposity measurements, specifically subcutaneous adipose tissue (SAT) and visceral adipose tissue (VAT), were assessed via ultrasonography (USG) with the Esaote MyLab™Eight eXP Ultrasound system, Genova, ITALY by a single experienced trained medical operator, as previously described [8]. Liver stiffness measurement (LSM) was conducted using transient elastography with the FibroScan^®^ system (Echosens, Paris, France). Measurement devices were inspected and calibrated in accordance with manufacturer guidelines.

### 2.3. Blood Sampling and Data Collection

Blood samples were collected at baseline and 18 months following the initiation of INSTI-based ART. After fractionation, plasma samples were stored at −80 °C. Plasma insulin levels were measured using the ALPCO^®^ commercial ELISA kit (Salem, NH, USA). Routine laboratory parameters, including glucose, triglycerides (TG), total cholesterol (TC), HDL-cholesterol (HDL-c), aspartate aminotransferase (AST), and alanine aminotransferase (ALT), were assessed. All measurements were taken under fasting conditions. Insulin resistance was evaluated using the Homeostatic Model Assessment for Insulin Resistance (HOMA-IR) index, with a cutoff value of >2.6 as proposed for the Mexican population [15].

### 2.4. Metabolite Extraction for Untargeted Metabolomics

Metabolite extraction was conducted at 4 °C. Plasma samples (100 µL) were thawed and mixed by vortexing with 800 µL of cold (−20 °C) LC-MS-grade methanol (JT Baker, Darmstadt, Germany) for 30 s. Samples were then incubated on ice for 30 min at 4 °C and centrifuged at 14,000× *g* for 10 min. The supernatant was collected and evaporated to dryness using a Savant DNA 120 SpeedVac vacuum concentrator. The resulting metabolite pellet was stored at −80 °C until analysis. On the day of analysis, the pellet was resuspended in 200 µL of acetonitrile/water (1:1) containing 0.1% formic acid, with 1 µL (1 ng/µL) of trans-zeatin added as an internal standard.

### 2.5. Untargeted Metabolomics

Metabolomic analysis was conducted using a Thermo Fisher Scientific™ Vanquish™ Flex UHPLC system (Bremen, Germany) coupled to a Thermo Fisher Scientific™ Orbitrap Exploris 120 mass spectrometer (Bremen, Germany). Samples (10 µL) were injected, and metabolites were separated on a C18 Kinetex column (1.7 µm, 150 × 2.1 mm; Phenomenex (Darmstadt, Germany) ) maintained at 30 °C. Mobile phase A consisted of water with 0.1% formic acid, and mobile phase B was acetonitrile/water (95:5) with 0.1% formic acid. Separation was performed at a flow rate of 0.2 mL/min with the following gradient: 1% B for 2 min, followed by an 8 min gradient from 1% B to 25% B, 12 min from 25% B to 85% B, 4 min from 85% B to 99% B (held for 4 min), and then a 2 min ramp from 99% B to 1% B (held for 3 min). Samples were randomized, and a quality control (QC) sample, prepared by pooling equal volumes of each sample, was injected every 8 samples to monitor mass spectrometer performance. Blank samples were also included.

Data acquisition was performed on a Thermo Fisher Scientific™ Orbitrap™ high-resolution accurate-mass (HRAM) mass spectrometer in data-dependent acquisition (DDA) mode using Thermo Fisher Scientific™ AcquireX software. Ionization was conducted via heated electrospray ionization (H-ESI) in positive (4.1 kV) and negative (2.5 kV) modes, with an ion transfer tube temperature of 350 °C, a vaporizer temperature of 250 °C, and nitrogen as the collision gas. Full scans were acquired at an Orbitrap resolution of 60,000 over a mass range of 70–1000 *m*/*z*. Data processing was performed using Thermo Fisher Scientific™ Compound Discoverer 3.3 SP3 software, which included background noise filtering, retention time correction, *m*/*z* identification (adduct assignment and *m*/*z* matching), peak detection, peak alignment, normalization, and chromatogram alignment. Data were filtered by area and peak rating >5. Metabolites were annotated using mass spectra fragmentation libraries (MS2), including mzCloud, ChemSpider, Human Metabolome Database (HMDB), KEGG, and PubChem.

### 2.6. Statistical Analysis of Clinical Dataset

The normality of continuous variables was assessed using the Kolmogorov–Smirnov test. Normally distributed variables were reported as mean ± standard deviation (SD), while non-normally distributed variables were expressed as median with interquartile range (IQR). Categorical variables were presented as absolute frequencies and percentages. Comparisons between cases and controls were conducted using Student’s *t*-test or the Mann–Whitney U test for continuous variables, and the χ^2^ test or Fisher’s exact test for categorical variables. Bivariate analyses were performed to estimate odds ratios (OR) with 95% confidence intervals (CI) for the association between independent variables and the outcome (weight gain, case group). Variables with a *p*-value < 0.05 or those deemed clinically relevant were included in a multivariable logistic regression model to calculate adjusted odds ratios (aOR) with 95% CI. A *p*-value < 0.05 was considered statistically significant. All statistical analyses were performed using SPSS v.31 (IBM Corp., Armonk, NY, USA).

### 2.7. Metadata Variables—Biplot

To identify key variables and discern patterns in the metabolomic data, Principal Component Analysis (PCA) and Random Forest classification were performed. Metabolic and clinical features were extracted from the dataset, with missing or infinite values imputed using the mean of each variable. PCA was conducted using the prcomp function and the vegan::rda method, with results visualized through biplots generated by the ggfortify and factoextra R v.4.1 packages [16,17,18]. Group classifications were overlaid using color coding based on a predefined categorical variable. For variable importance, a Random Forest model was fitted using the randomForest package with 1000 trees and default parameters. Variable importance was evaluated using the MeanDecreaseGini metric and visualized with bar plots. All statistical analyses and visualizations were performed in R (version 4.5.0).

### 2.8. Multivariate Analysis

Partial Least Squares Discriminant Analysis (PLS-DA) was performed using the mixOmics package in R [19]. The input dataset comprised a transposed matrix of metabolite abundances, preprocessed by converting all values to numeric values and assigning sample group labels based on predefined classifications. The PLS-DA model was fitted with two components (ncomp = 2) to capture the primary sources of variance associated with group differentiation. Explained variance for each component was calculated and used to annotate the axes of the resulting score plot. Group separation and 95% confidence ellipses were visualized using ggplot2 [20], with customized color schemes and wsthetic adjustments to enhance clarity. This approach facilitated clear class discrimination in the dataset, serving as a foundation for subsequent multivariate comparisons. A total of 2015 annotated metabolites were analyzed.

To further explore group separation in metabolomic profiles, multivariate statistical analyses were conducted in R (version 4.5.0) [16,18]. PLS-DA was performed using the mixOmics package [21], retaining two principal components to maximize group separation and visualize sample clustering. The explained variance of each component was extracted to assess the model’s discriminatory power. Additionally, Principal Coordinate Analysis (PCoA) was conducted on a Bray–Curtis distance matrix using the vegan [21] and ape packages [22], providing an unsupervised representation of compositional dissimilarities between groups. Group significance was evaluated using PERMANOVA (adonis2) [23] and pairwise comparisons to statistically validate clustering patterns.

### 2.9. Metabolite Classification and Filtering

Metabolite classification was conducted using PubChem identifiers (CID, InChIKey) and chemical taxonomies retrieved via the classyfireR package in R [24]. Each compound was assigned to a superclass category to enable systematic classification into major biochemical families. To prioritize biologically relevant metabolites, compounds were filtered to include only those belonging to the following superclasses: alkaloids and derivatives (associated with anti-inflammatory and antioxidant activities), lipids and lipid-like molecules, organic acids and derivatives (including amino acids, peptides, and their derivatives), organic oxygen compounds (primarily carbohydrates), and phenylpropanoids and polyketides (recognized for their anti-inflammatory and antioxidant properties). Following this filtering step, approximately 789 metabolites were retained for subsequent analyses.

### 2.10. Heatmaps

Metabolomic profiles were analyzed in R (version 4.5.0) to generate comparative heatmaps. A curated list of metabolites was used to filter the normalized abundance matrix, retaining only compounds of biological relevance. Raw abundance values were log_2_-transformed (log_2_(x + 1))(\log_2_(x + 1))(log_2_(x + 1)) to reduce skewness and stabilize variance across samples. Heatmaps were created using the pheatmap package [25], employing hierarchical clustering with Euclidean distance and complete linkage for both metabolites and samples. The visualization strategy included heatmaps of all individual samples, with row scaling to emphasize relative abundance patterns. Experimental design metadata were integrated as column annotations to facilitate the visualization of condition-specific clustering patterns.

### 2.11. Volcano Plots

Differential abundance analysis was conducted in the R/Bioconductor environment. Raw count data were filtered to remove metabolites with low relative abundance using the edgeR package [26], applying the filterByExpr function to retain features with sufficient counts across conditions. Normalization and statistical modeling were performed using DESeq2 [27], which estimates size factors, dispersions, and fits negative binomial generalized linear models. For exploratory comparisons, the limma package with the voom transformation [28] was used to stabilize mean–variance relationships and enable linear modeling. To account for multiple testing, *p*-values were adjusted using the Benjamini–Hochberg false discovery rate (FDR) procedure. Metabolites were deemed significantly differentially abundant if they met both criteria: an absolute fold change of at least 1.5 (|log_2_FC| ≥ 1.5) and an adjusted *p*-value (FDR) < 0.05. Results were visualized using volcano plots generated with the EnhancedVolcano package [29], providing an intuitive representation of fold change versus an adjusted *p*-value, with statistically significant features highlighted. The resulting set of differentially abundant metabolites was used for downstream analyses, including data visualization, functional enrichment, and pathway exploration, to identify the biological processes most impacted under the studied conditions.

### 2.12. Enrichment Pathway

Metabolite set enrichment analysis was performed using MetaboAnalyst 6.0 [30]. Compound names were standardized to ensure compatibility with reference libraries. Only well-annotated compounds from the Human Metabolome Database (HMDB) present in integrated pathway libraries were mapped. Greek letters were replaced with their English equivalents (e.g., “alpha,” “beta”), and ambiguous cases were resolved using the Compound ID Conversion tool. Exact matches were automatically assigned, while compounds without unique matches were manually curated using approximate searches and KEGG ID verification.

Enrichment analysis was conducted using the globaltest algorithm, which employs a generalized linear model to evaluate associations between metabolite sets and the experimental outcome. A Q-statistic was calculated for each metabolite set as the average of squared covariances (Q values) between individual metabolites and the outcome variable. This method has demonstrated performance comparable or superior to other enrichment approaches.

Pathway libraries were sourced from RaMP-DB, integrating data from HMDB, KEGG, Reactome, and WikiPathways, covering 3694 metabolite and lipid pathways. To reduce biases from incomplete metabolome coverage, enrichment results were interpreted relative to the platform-specific reference metabolome. Significant metabolite sets were identified and visualized using dotplots, which intuitively depict the degree of enrichment and statistical significance across pathways.

## 3. Results

### 3.1. Characteristic of the Study Population

This longitudinal study, nested within the MICTLAN cohort, included 66 male participants (28 controls and 38 cases). The mean age was 27.7 years, with 60.6% of participants classified as HIV stage 2 per CDC criteria. Approximately 48% of participants received bictegravir/tenofovir alafenamide/emtricitabine (BIC/TAF/FTC), and 51% received dolutegravir/abacavir/lamivudine (DTG/ABC/3TC) as their INSTI-based regimen. No significant differences in weight gain were observed according to the NRTI backbone (*p* = 0.793), suggesting no synergistic effect between BIC and TAF in this cohort.

At baseline, body composition assessed by electrical bioimpedance (TANITA FitScan BC-545F) showed no significant differences between groups in muscle mass, body fat, body water, or bone mass. Similarly, baseline measurements of subcutaneous adipose tissue (SAT) and visceral adipose tissue (VAT) via ultrasonography, as well as liver stiffness via transient elastography (FibroScan^®^), were comparable between groups. Laboratory parameters, including glucose, lipid profile, insulin, HOMA-IR, liver function tests (aspartate aminotransferase [AST], alanine aminotransferase [ALT]), CD4+ and CD8+ T-cell counts, showed no significant differences at baseline, although a trend towards higher triglyceride levels was observed in the case group. Although baseline gamma-glutamyl transferase (GGT) levels differed between groups (*p* < 0.05), these values remained within the normal range. In addition, HIV RNA viral load was significantly higher in the case group (*p* = 0.001). Baseline characteristics are summarized in Table 1.

**Table 1:** Clinical and basal characteristics of participants.

Characteristics	General (N = 66)	Controls (N = 38)	Cases (N= 28)	*p*
Age (years)	27.74 ± 7.09	26.84 ± 6.49	28.96 ± 7.78	0.261
Body weight (kg)	68.04 ± 11.52	67.48 ± 9.46	67.30 ± 13.63	0.661
BMI (kg/m^2^)	23.36 ± 3.40	23.50 ± 2.81	23.16 ± 4.3	0.691
HIV RNA VL (copies/mL)	35,879 [8740–101,317]	21,614 [4228–50,314]	68,258 [33,149–233,295]	0.001
Log VL	4.41 ± 0.93	4.18 ± 0.92	4.74 ± 085	0.678
T CD4+ (cel/μL)	279.23 ± 155.75	298.90 ± 174.96	252.53 ± 123.16	0.269
T CD8+ (cel/μL)	665.74 ± 570.66	561.32 ± 447.621	807.45 ± 687.92	0.436
HIV stage CDC				
1	7.6% (5/66)	10.5% (4/38)	3.6% (1/28)	
2	60% (40/66)	65.8% (25/38)	53.6% (15/28)	0.193
3	31.8% (21/66)	23.7% (9/38)	42.9% (12/38)	
INSTI				
BIC	48.48% (32/66)	50% (19/38)	53.57% (15/28)	0.808
DTG	51.51% (34/66)	50% (19/38)	46.42% (13/28)	
Insulin (µUI/mL), median [IQR]	12.69 [10.34–17.8)	12.38 [9.83–18.27]	12.89 [11.01–16.65]	0.683
HOMA-IR, median [IQR]	1.2 [0.7–2.02]	1.1 [0.6–2.32]	1.3 [0.92–1.17]	0.399
Muscle mass (%), median [IQR]	35.85 [34.52–39.05]	36.55 [34.7–39.05]	35.5 [34.5–38.8]	0.590
Body fat (%)	21.24 ± 4.12	20.91 ± 3.88	21.69 ± 4.46	0.460
Body water (%)	50.21 ± 3.73	50.32 ± 3.60	50.05 ± 3.94	0.451
Bone (kg)	2.86 ± 0.35	2.79 ± 0.28	2.96 ± 0.41	0.065
SAT (cm)	1.73 ± 0.80	1.80 ± 0.83	1.64 ± 0.77	0.423
VAT (cm)	3.73 ± 0.87	3.70 ± 0.83	3.76 ± 0.94	0.801
Liver stiffness (kPa)	4.69 ± 0.93	4.6 ± 0.93	4.82 ± 0.93	0.681
TGO (AST) (UI/L)	30.27 ± 6.31	30.13 ± 8.45	28.35 ± 10.37	0.377
TGP (ALT) (UI/L)	29.37 ± 9.285	30.42 ± 5.51	30.07 ± 7.35	0.474
GGT (UI/L) median [IQR]	40 [31.5–45.75]	43.5 [33–56]	33 [29.6–42.33]	0.012
PA (UI/L)	69 [56–89]	75.2 [56–88.14]	65.2 [52–84.12]	0.259
TC (mg/dL)	169.25 ± 14.61	164.05 ± 12.79	176.32 ± 14.12	0.436
TG (mg/dL)	146.43 ± 13.26	141.07 ± 12.55	153.71 ± 10.60	0.078
HDL (mg/dL), median (IQR)	23 [19–32]	23.14 [19.12–32.4]	24.25 [20.5–28.8]	0.607
Creatinine mg/dL, median (IQR)	0.89 (0.78–0.98)	0.89 (0.78–0.98)	0.92 (0.81–0.98)	0.927

Data are presented as mean (SD) or n (%) or median [IQR]. Abbreviations: BMI: Body Mass Index; INSTI: integrase strand transfer inhibitors; HOMA-IR: Homeostatic Model Assessment for Insulin Resistance; kPa: kilopascal; SAT: subcutaneous adipose tissue; VAT: visceral adipose tissue; VL: viral load; log VL: logarithm viral load; IQR: interquartile range; BIC: bictegravir; DTG: dolutegravir; TGO or AST: alanine aminotransferase; TGP or ALT: apartate transaminase; GGT: gamma-glutamyl transferase; PA: alkaline phosphatase; TC: total cholesterol; TG: triglycerides; HDL: high-density lipoprotein.

After 18 months of INSTI-based ART, participants were classified based on weight gain: cases exhibited weight gain > 10% of baseline weight, while controls showed no significant weight increase (75.16 ± 15.20 kg vs. 67.48 ± 9.46 kg; *p* < 0.05). Insulin resistance, defined as HOMA-IR > 2.6, was significantly higher in cases compared to controls (*p* < 0.05). Cases also displayed greater VAT thickness (3.85 cm [IQR 3.27–4.72] vs. 3.40 cm [IQR 2.90–3.88]; *p* = 0.009) and elevated triglyceride levels (162.50 mg/dL [IQR 148.25–171.25] vs. 145.00 mg/dL [IQR 136.50–152.00]; *p* = 0.004). Although liver stiffness differed significantly between cases and controls (*p* = 0.034), values remained within normal parameters. Results at 18 months are presented in Table 2.

**Table 2:** Clinical characteristics of participants at 18 months post-ART with INSTI.

Characteristics	General (N = 66)	Controls (N = 38)	Cases (N = 28)	*p*
Body weight (kg)	70.7 ± 12.7	67.48 ± 9.46	75.16 ± 15.20	0.023
T CD4+ (cel/μL)	754 [613–932]	772.50 [636.05–956.50]	668.15 [566.75–859.50]	0.102
T CD8+ (cel/μL)	1103 [867–1444]	1129 [903–1445]	1092 [829–1444]	0.456
TCD4+/TCD8+ ratio	0.64 [0.49–0.87]	0.61 [0.55–0.88]	0.67 [0.49–0.85]	0.886
Insulin (µUI/mL)	12.64 [9.66–16.75]	11.61 [8.16–15.53]	13.57 [11.28–24.38]	0.179
HOMA-IR > 2.6	25 (37.87%)	10 (15.15%)	15 (22.72%)	0.049
Muscle mass (%)	37.55 [34.85–41.18]	38.85 [35.32–41.37]	35.85 [34.3–38.55]	0.073
Body fat (%)	21.79 ± 4.76	21.23 ± 3.91	22.52 ± 5.71	0.308
Body water (%)	49.90 [46.80–53.75]	51.80 [48.30–53.88]	47.55 [46.08–50.80]	0.053
Bone (kg)	2.86 ± 0.35	2.78 ± 0.28	2.95 ± 0.41	0.065
SAT (cm)	1.85 [1.40–2.50]	1.75 [1.30–2.58]	2.05 [1.55–2.50	0.583
VAT (cm)	3.60 [3.02–4.38]	3.40 [2.90–3.88]	3.85 [3.27–4.72]	0.009
Liver stiffness (kPa)	4.50 [4.00–4.90]	4.7 [4.0–5.32]	4.3 [3.82–4.67]	0.034
TGO (AST) (UI/L)	36 [30–40]	35 [27–41]	37 [35–40]	0.216
TGP (ALT) (UI/L)	32.86 ± 10.15	33.68 ± 10.57	31.75 ± 9.60	0.441
GGT (UI/L)	24 [21–26]	24.5 [21.0–26.0]	24.5 [22.5–26.0]	0.583
PA (UI/L)	64.50 [61–72]	64.50 [61.00–72.00]	66.50 [61.00–72.00	0.995
TC (mg/dL)	135 [89–158]	135.00 [89.25–157.00]	135.50 [89.00–177.50]	0.617
TG (mg/dL)	149.50 [141.25–159.50]	145.00 [136.50–152.00]	162.50 [148.25–171.25]	0.004
HDL (mg/dL)	41 [29–45]	34 [28–45]	42 [31–44]	0.580
Creatinine mg/dL	0.89 (0.78–0.98)	0.89 (0.78–0.98)	0.92 (0.81–0.98)	0.927

Data are presented as mean (SD) or n (%) or median [IQR]. Abbreviations: HOMA-IR: Homeostatic Model Assessment for Insulin Resistance; kPa: kilopascal; SAT: subcutaneous adipose tissue; VAT: visceral adipose tissue; IQR: interquartile range; BIC: bictegravir; DTG: dolutegravir; TGO or AST: alanine aminotransferase; TGP or ALT: aspartate transaminase; GGT: gamma-glutamyl transferase; PA: alkaline phosphatase; TC: total cholesterol; TG: triglycerides; HDL: high-density lipoprotein.

### 3.2. Factors Associated with Weight Gain

Bivariate analysis revealed significant associations between weight gain and several factors. Insulin resistance, defined as HOMA-IR > 2.6, was associated with weight gain (OR 3.23; 95% CI 1.14–9.10; *p* = 0.023). Similarly, visceral adipose tissue (VAT) thickness > 4 cm (OR 4.50; 95% CI 1.60–13.03; *p* = 0.004), triglyceride levels >150 mg/dL (OR 3.90; 95% CI 1.38–10.94; *p* = 0.008), HIV RNA viral load >100,000 copies/mL mL (OR 3.45; 95% CI 1.08–10.96; *p* = 0.0002), and HIV RNA viral load > 50,000 copies/mL mL (OR 8.05; 95% CI 2.65–24.43; *p* = 0.035) were significantly associated with weight gain. These findings are summarized in Table 3.

**Table 3:** Association of indicators and weight gain in PLW.

Characteristics	Controls (N = 38)	Cases (N = 28)	OR (95% CI)	*p*
HOMA-IR > 2.6	10 (15.15%)	15 (22.72%)	3.23 (1.14–9.10)	0.023
VAT ≥ 4 cm	12 (18.18%)	19 (28.78%)	4.50 (1.60–13.03)	0.004
TG ≥ 150 mg/dL	12 (18.18%)	18 (27.27%)	3.90 (1.38–10.94)	0.008
Baseline HIV RNA VL > 50,000 copies/mL	9 (13.63%)	20 (30.30%)	8.05 (2.65–24.43)	0.0002
Baseline HIV RNA VL > 100,000 copies/mL	6 (9.09%)	11 (16.66%)	3.45 (1.08–10.96)	0.035

Abbreviations: OR: odds ratio; CI: confidence interval; HOMA-IR: Homeostatic Model Assessment for Insulin Resistance; VAT: visceral adipose tissue; TG: triglycerides.

In the multivariate analysis, HOMA-IR > 2.6 was not independently associated with weight gain at 18 months in the cases group (aOR 2.37; 95% CI 0.72–12.67; *p* = 0.155). However, visceral adipose tissue (VAT) thickness >4 cm (aOR 5.77; 95% CI 1.52–21.87; *p* = 0.010), triglyceride levels >150 mg/dL (aOR 3.82; 95% CI 1.04–13.98; *p* = 0.042), and baseline HIV RNA viral load >50,000 copies/mL (aOR 6.58 (1.83–23.58); *p* = 0.004) emerged as strong independent predictors of weight gain. These results are summarized in Table 4. Importantly, these associations were also maintained in the multivariate model considering baseline viral load > 100,000 copies/mL (Table S1), showing that HIV RNA was identified as a baseline predictor of weight gain.

**Table 4:** Multivariate logistic regression of metabolic predictors of weight gain.

Characteristics	aOR (95% CI)	*p*
HOMA-IR > 2.6	1.90 (0.51–7.07)	0.334
VAT ≥ 4 cm	5.77 (1.52–21.87)	0.010
TG ≥ 150 mg/dL	3.82 (1.048–13.98)	0.042
Baseline HIV RNA VL ≥ 50,000 copies/mL	6.58 (1.83–23.58)	0.004

Abbreviations: OR: odds ratio; CI: confidence interval; HOMA-IR: Homeostatic Model Assessment for Insulin Resistance; VAT: visceral adipose tissue; VL: viral load; TG: triglycerides.

Biplot PCA Analysis (Figure S2A) and Random Forest Analysis (Figure S2B) were used to assess the relative contributions of clinical variables for group classification. As summarized in Figure S1, the insulin resistance measured by HOMA-IR contributes to the segregation of analyzed samples.

### 3.3. Plasma Metabolites in the Cases and Controls Groups Pre- and Post- INSTI-ART Are Separated in Distinct Clusters

**Figure 1:**
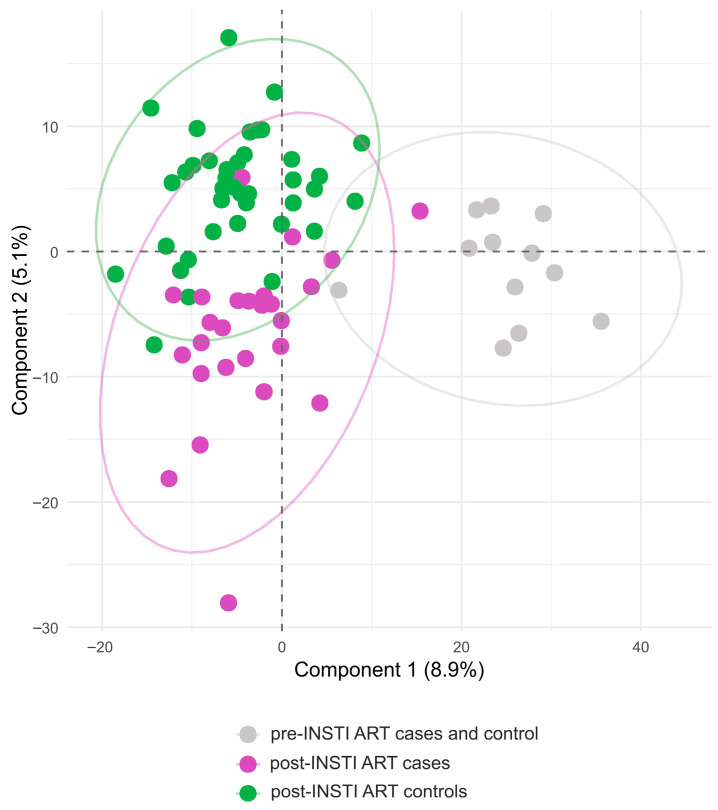
Partial Least Squares Discriminant Analysis (PLS-DA) of metabolomic profiles at baseline, cases, and controls. The model demonstrates partial separation between cases (purple) and controls (green) after 18 months of INSTI-based treatment, whereas baseline samples (gray) cluster independently.

The metabolites that contributed the most to the observed group separation were identified by pairwise PLS-DA comparisons. PLS-DA analysis was conducted in the positive and negative mode. Three distinct clusters representing all PWH participants pre-INSTI ART, control PWH post-INSTI ART (with weight gain <10% baseline weight gain), and cases PWH post-INSTI ART (with weight gain > 10% baseline weight gain) were identified. The comparison between controls and cases post-INSTI ART tends to exhibit intragroup aggregation and intergroup dispersion, suggesting that the samples of both groups were different from their counterpart at the baseline ART (Figure 1).

### 3.4. Differential Metabolite Analysis

The top 50 metabolites from control and case groups were firstly selected and analyzed in hierarchical cluster (heatmap) comparing top 50 metabolites from plasma samples pre- and post-INSTI treatment (Figure S3A) and the respective volcano plot displaying significant fold change in each metabolite (Figure S3B).

**Figure 2:**
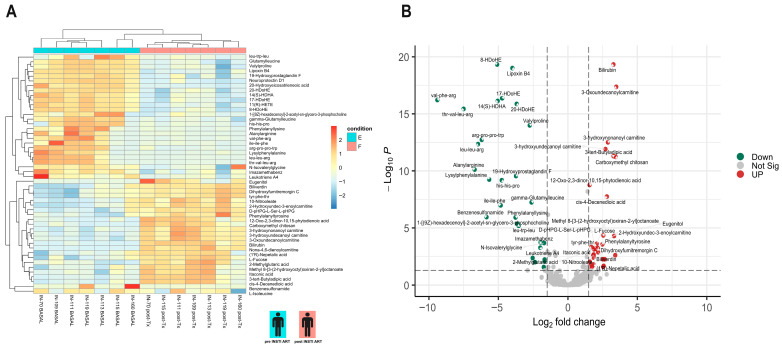
Metabolite analysis of control group. (**A**). Heatmap of differential metabolites in the control group before (pre-ART, E) and after antiretroviral therapy (post-ART, F). Prior to ART, patients exhibited enrichment in acylcarnitines (3-hydroxynonanoyl, 3-hydroxyundecanoyl, 3-oxoundecanoyl, and Nona-4,6-dienoylcarnitine) and bilirubin, consistent with a pro-oxidative state and lipid metabolism dysfunction. In contrast, after 18 months of ART, lipid mediators with anti-inflammatory and pro-resolving functions, including lipoxin B4, neuroprotectin D1, prostaglandin derivatives, and HDHAs, predominated, reflecting a transition toward a more regulatory and inflammation-resolving metabolic profile. (**B**). Volcano plot of differential metabolites in the control group before and after ART. Post-ART analysis showed increased levels of L-isoleucine, bilirubin, and multiple acylcarnitines, whereas anti-inflammatory and pro-resolving lipid mediators (lipoxins, neuroprotectins, HDoHE, HDHA, HETEs) and several amino acid-derived peptides were reduced.

The top 50 metabolites from control and case groups were selected and hierarchical cluster analysis (heatmap) was performed (Figure 2A), and the significant contribution of each metabolite was determined using a volcano plot (Figure 2B). Thus, the acylcarnitines (3-hidroxynonanoyl carnitine, 3-hidroxyundecanoyl carnitine, 3-Oxo undecanoyl carnitine, 2-hidroxyundec-3-enoyl carnitine, 6-hidroxynon-2-enoyl carnitine and nona-4,6-dienoylcarnitine) have high relative abundance in both groups, whereas docosahexanoic acid (DHA)-derivate oxylipins such as 18-H DoHE, 11R-H DoHE 14-H DoHE, 17-H DoHE, and 20-H DoHE, lipid mediators with anti-inflammatory and pro-resolving functions (lipoxins, neuroprotectins, HDHAs, and HDoHEs), were in low relative abundance, suggesting that relative abundance of these metabolites represents an effect of the INSTI-ART. After 18 months of INSTI-ART, the control group showed relative abundance of itaconic acid and medium-chain fatty acids such as cis-4-decenedioic acid (Figure 2A,B).

**Figure 3:**
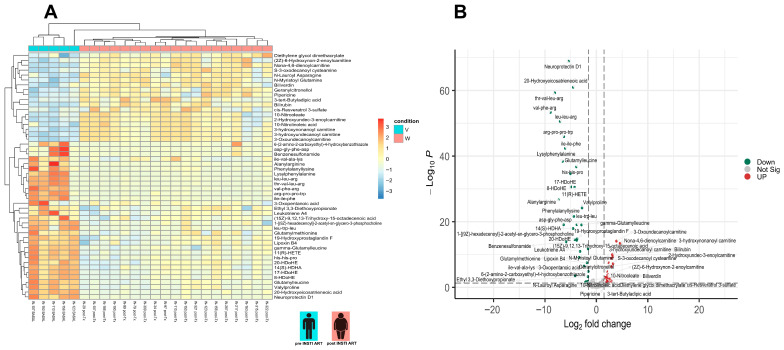
Metabolite analysis of case group. (**A**). Heatmap of differential metabolites in the case group before (V) and after ART (W). The pre-ART state was characterized by higher abundance of acylcarnitines and bilirubin, whereas the post-ART state showed a predominance of lipid mediators with anti-inflammatory and pro-resolving functions (lipoxins, neuroprotectins, HDHAs, HDoHEs). (**B**). Volcano plot of differential metabolites in the case group before and after ART. Post-ART analysis showed a significant increase in acylcarnitines and bilirubin, whereas anti-inflammatory and pro-resolving metabolites (lipoxins, neuroprotectins, HDoHEs, HDHAs, HETEs) and amino acid-derived peptides were reduced.

Hierarchical clustering analysis (heatmap) revealed distinct metabolic separation between cases and controls after 18 months of INSTI-based therapy (Figure 3A). In the case group (participants with weight gain > 10%), there was a higher relative abundance of medium-chain acylcarnitines, bilirubin, and amino acid derivatives (e.g., gamma-glutamyl leucine, glutamylmethionine, glutamyl leucine, valylproline, and alanylarginine). Conversely, the control group exhibited a metabolic profile enriched in lipid mediators with anti-inflammatory and pro-resolving functions, including lipoxins, neuroprotectins, HDHAs, HDoHEs, and itaconic acid. The volcano plot (Figure 3B) confirmed these differences, highlighting significant overexpression of acylcarnitines (C9–C13) and bilirubin in cases, contrasted with a relative reduction in polyunsaturated fatty acid-derived metabolites with pro-resolving and immunomodulatory functions (e.g., neuroprotectin D1, austroinulin, 20-HDoHE, 14(S)-HDHA, 11(R)-HETE, and lipoxin B4) in controls. Additionally, the case group showed increased relative abundance of S-3-oxodecanoyl cysteamine, a fatty acyl thioester. These findings suggest that, following INSTI exposure, cases undergo metabolic reprogramming associated with mitochondrial dysfunction and oxidative stress, while controls maintain pro-resolving and anti-inflammatory responses, potentially explaining differences in susceptibility to weight gain.

### 3.5. KEGG Analysis Reveals Divergent Pathway Signatures

**Figure 4:**
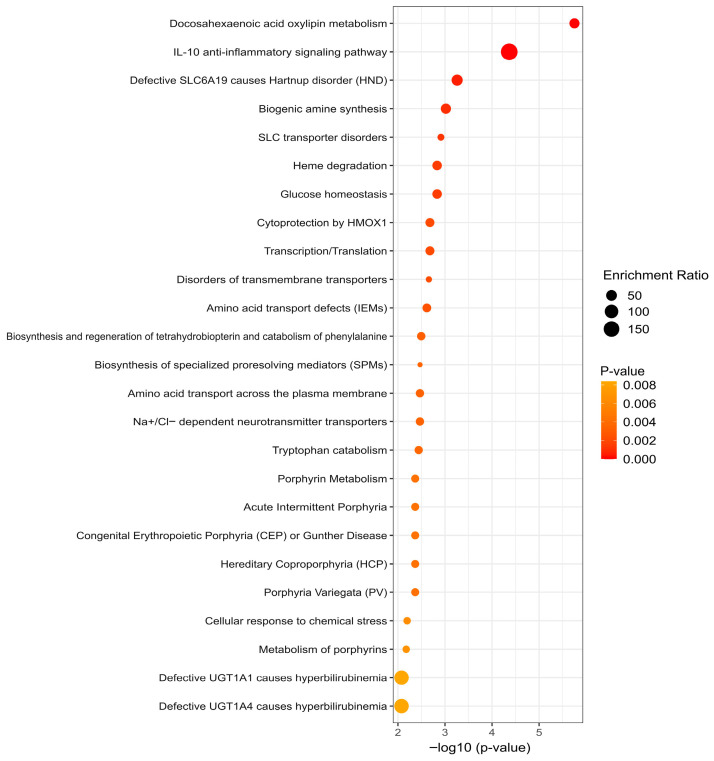
Enrichment pathway analysis in control group. Top 25 KEGG pathway enrichments with *p* value are shown. Glucose homeostasis, amino acid transport pathways, biosynthesis of specialized pro-resolving lipid mediators (SPMs), and dysfunction of SLC transporters with emphasis on the SLC6A19 are displayed. The *y*-axis represents the name of the pathway, and the *x*-axis represents the *p* value, the degree of KEGG pathway enrichment.

**Figure 5:**
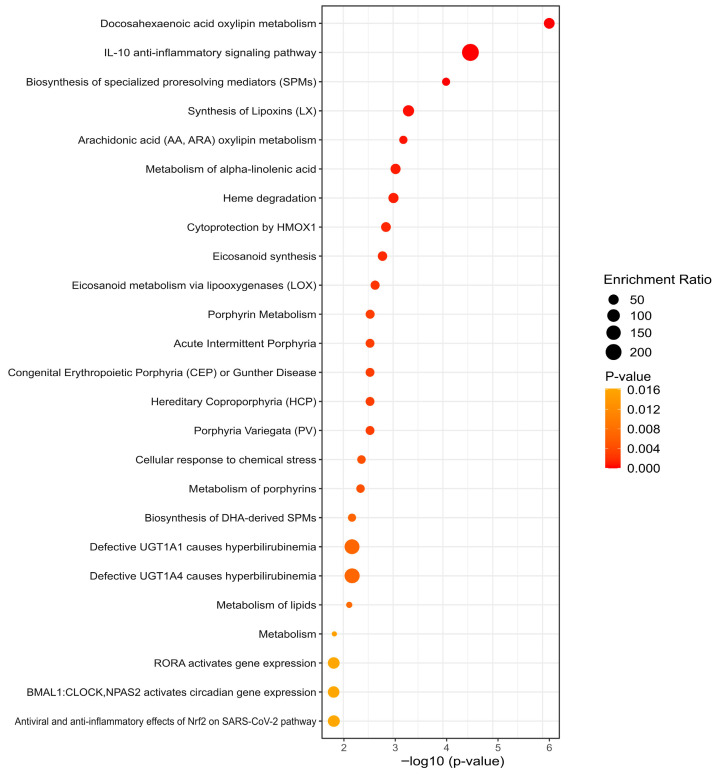
Enrichment pathway analysis in the case group. Top 25 KEGG pathway enrichments with *p* value are shown. RORA and circadian (BMAL1:CLOCK) signaling were displayed, suggesting a metabolic adaptation oriented toward homeostasis, inflammation control, preservation of energy balance, and disruption in the signaling of circadian cycle as part of worse metabolic adaptation post-INSTI ART. Similarly to the control group, the predominant enrichment of pathways linked to polyunsaturated fatty acid metabolism and metabolism docosahexanoic acid (DHA), together with the activation of anti-inflammatory (IL-10) and biosynthesis of specialized pro-resolving lipid mediators (SPMs), was also displayed. The *y*-axis represents the name of the pathway, and the *x*-axis represents the *p* value, the degree of KEGG pathway enrichment.

Comparative metabolomic analysis following antiretroviral therapy (ART) revealed distinct metabolic profiles between controls and cases with weight gain. In controls, enriched pathways were primarily associated with polyunsaturated fatty acid metabolism, particularly the biosynthesis of specialized pro-resolving lipid mediators (SPMs), as well as glucose homeostasis and amino acid transport. These findings suggest a more effective metabolic adaptation to integrase strand transfer inhibitor (INSTI)-based ART, characterized by improved inflammation control and maintained energy balance (Figure 4). In contrast, cases displayed a broader and more heterogeneous metabolic profile, marked by heightened activation of lipid metabolism pathways, including significant accumulation of acylcarnitines and enhanced metabolism of alpha-linolenic acid (a precursor of DHA). Additionally, pathway enrichment analysis in people with HIV (PWH) in the case group revealed activation of Retinoic Acid-related Orphan Receptor alpha (RORA) and circadian (BMAL1:CLOCK) signaling, indicating a metabolic adaptation aimed at homeostasis, inflammation control, and energy balance, but accompanied by disrupted circadian signaling, suggestive of poorer metabolic adaptation post-INSTI ART. Furthermore, the prominence of arachidonic acid and oxylipin metabolism, alongside IL-10 anti-inflammatory pathways, points to a persistent inflammatory state with inadequate pro-resolving activity (Figure 5). Overall, these differences highlight distinct molecular mechanisms: controls exhibited a regulatory metabolic profile, whereas cases showed evidence of metabolic dysfunction. 

## 4. Discussion

Integrase strand transfer inhibitors (INSTIs) are generally safe with minimal drug interactions, but weight gain beyond the expected return-to-health effect has emerged as a significant concern [1,4]. Our analysis highlights potential links between weight gain and first-line INSTI-based antiretroviral therapy (ART) regimens (dolutegravir [DTG] and bictegravir [BIC]), focusing on glucose and lipid metabolism. Weight gain exceeding 10% from baseline at 18 months was used as a clinical criterion to effectively distinguish cases from controls in our cohort. Factors associated with weight gain in people with HIV (PWH) on INSTI-ART include female sex, older age, ethnicity, genetics, and low baseline CD4+ T-cell counts and HIV viral load > 100,000 copies/mL [4,31,32,33,34,35]. Unlike with Hill et al. (2024) [31], baseline CD4+ T-cell counts were similar between groups in this cohort, suggesting that low CD4 counts (<200 cells/uL) may not necessarily be a baseline predictor of weight gain. On the other hand, previous studies have reported pronounced weight gain following treatment initiation, mainly observed in PWH with advanced HIV disease and HIV viral load > 100,000 copies/mL, which can be explained by the deleterious effect of HIV on adipose tissue morphology, function, and metabolism [9,34]. In this regard, weight gain observed in the case group from this study was strongly associated even with HIV viral load > 50,000 copies/mL. Future studies are necessary to validate this cutoff value as a baseline marker predictive of excessive weight gain in PWH on INSTI-based regimens. On the other hand, use of baseline HIV viral load as a predictive marker for weight gain, could guide clinicians in the selection of first-line ART between INSTI or NNRTI. In this regard, the NNRTI doravirine has been recently recommended as first-line treatment for HIV to have a better lipid profile and little impact on weight gain [36,37]. The safety profile (in terms of minimal weight gain) of Doravirine as first-line treatment in patients with baseline viral loads > 50,000 copies/mL requires further investigation.

Older age is a known risk factor for impaired glucose metabolism, particularly in PWH on INSTI-based regimens [38]. In this study, PWH had a mean age of 28 years, slightly older in cases than controls. Insulin resistance at 18 months post-INSTI ART was associated with weight gain but was not a predictor of this outcome. PWH remained normoglycemic throughout the 18-month follow-up. However, significant changes in fasting blood glucose in PWH on INSTIs typically occur at three years, with a cumulative type 2 diabetes mellitus (T2DM) incidence of 6.7% [39]. Notably, glucose homeostasis pathways were enriched only in the control group, suggesting clinicians should monitor the HOMA-IR index rather than fasting glucose in PWH on INSTI regimens [38].

Visceral adipose tissue (VAT) expansion has been documented within 24 months of initiating DTG-based regimens [4]. In this study, VAT thickness > 4 cm and hypertriglyceridemia at 18 months post-INSTI ART were associated with weight gain and served as predictors in the analyzed PWH. Although VAT biopsies were not available, the increased VAT thickness in cases likely reflects adipocyte hypertrophy and insulin resistance, as previously reported [9,10]. Hypertrophic adipocytes in obesity often fail to store excess lipids in droplets, leading to toxic levels of circulating fatty acids in non-adipose tissues [40,41].

Using a circulating metabolomics approach in 66 PWH, we identified distinct metabolic profiles between those with weight gain and those achieving return-to-health. Metabolites were filtered based on their relevance to metabolism, inflammation, and obesity. The metabolomic analysis revealed a significant acylcarnitine signature in PWH with weight gain, characterized by elevated plasma medium-chain acylcarnitines. Acylcarnitine formation is critical for glucose homeostasis, and impaired short-chain acylcarnitine production is associated with reduced glucose disposal in obesity and coronary artery disease [42,43]. Accumulation of fatty acids and carnitine derivatives has been observed in patients with metabolic decompensation and disrupted mitochondrial homeostasis [44]. Inefficient fatty acid utilization as an energy source and specific acylcarnitine accumulation can disrupt lipid homeostasis, leading to dysregulated mitochondrial fatty acid oxidation [45]. Elevated levels of metabolites indicative of dysregulated fatty acid oxidation are also seen in T2DM patients with subsequent major adverse cardiovascular events [46], underscoring the role of mitochondrial dysfunction post-INSTI ART initiation.

Oxylipin and docosahexaenoic acid (DHA) metabolism pathways were prominent in both groups. These bioactive molecules, derived from polyunsaturated fatty acids (PUFAs) such as arachidonic acid (AA; 20:4), eicosapentaenoic acid (EPA; 20:5), DHA (22:6), and linoleic acid (LA; 18:2), are linked to atherosclerosis, endothelial dysfunction, myocardial dysfunction, and immune cell and fibroblast overactivation [47]. The role of PUFAs in adipose tissue fibrosis under INSTI exposure warrants further investigation [9,10]. Notably, circulating DHA is positively associated with cognitive function during aging and inversely related to cognitive decline [48]. Future studies should explore DHA levels and cognitive impairment in PWH on INSTI-ART, independent of weight gain.

In addition to acylcarnitine accumulation, PWH with weight gain exhibited higher relative abundance of circulating amino acid derivatives (glutamylmethionine, gammaglutamyl leucine, glutamyl leucine, valylproline, and alanylarginine) compared to controls. Elevated gammaglutamyl leucine levels are associated with increased cardiometabolic risk [49]. Enrichment pathway analysis revealed dysfunction of SLC transporters, particularly SLC6A19, in controls. Amino acids modulate glucose homeostasis, and their availability is regulated by transporters like SLC6A19, which enhance insulin secretion and signaling in various tissues [50,51]. These findings suggest SLC6A19 contributes to amino acid homeostasis in PWH without weight gain post-INSTI ART.

Bailin et al. (2025) described adipose tissue reprogramming in virologically suppressed PWH, driving an accelerated cardiometabolic state [11]. While these findings confirm metabolic reprogramming post-INSTI ART, their cross-sectional design limits causal inferences. Our longitudinal study suggests mitochondrial dysfunction contributes to metabolic signatures resembling metabolic syndrome [52]. Mitochondrial dysfunction has also been reported with other antiretroviral drug classes [53,54]. Our results indicate INSTI use is associated with mitochondrial dysfunction and altered anti-inflammatory mediators. Since mitochondrial dysfunction also occurs in skeletal muscle, further studies are needed to elucidate its role in metabolic syndrome and sarcopenic obesity in PWH [55].

Although both PWH groups showed enrichment of liver function-related pathways (heme degradation, porphyrin metabolism), no evidence of liver damage was observed. However, the Retinoic Acid-related Orphan Receptor alpha (RORA) pathway was enriched only in PWH with weight gain post-INSTI ART. RORA, a nuclear receptor superfamily member, links inflammatory and metabolic signaling, and its overexpression in VAT may contribute to dysfunction and insulin resistance in obesity [56]. Additionally, enrichment of the BMAL1:CLOCK pathway suggests disrupted circadian signaling, indicative of poorer metabolic adaptation post-INSTI ART [57].

## 5. Conclusions

We utilized nontargeted plasma metabolomics to characterize metabolic profiles associated with weight gain in people with HIV (PWH) after 18 months of integrase strand transfer inhibitor (INSTI)-based antiretroviral therapy (ART) as first-line treatment. Approximately 700 differential metabolites were identified in plasma from PWH. Our findings highlight the accumulation of circulating medium-chain acylcarnitines, indicative of mitochondrial dysfunction, alongside insulin resistance (assessed by HOMA-IR). Overweight and obesity secondary to INSTI-ART warrant further investigation, as specific treatments for these outcomes are currently unavailable for PWH. These results emphasize the need for targeted metabolomic studies focusing on acylcarnitines, amino acid homeostasis, related pathways, and their associations with INSTI use.

### 5.1. Study Strengths

This study has several strengths. The inclusion of HOMA-IR index assessments, tailored to the specific population, enhanced the evaluation of insulin resistance. The longitudinal design enabled the identification of metabolites as biomarkers of insulin resistance. Despite the small sample size in this pilot study, case–control assignment was based on distinct phenotypic extremes. Additionally, the use of plasma as a minimally invasive liquid biopsy facilitated metabolomic analysis.

### 5.2. Study Limitations

This study faced several limitations. Loss to follow-up occurred due to patients losing social security coverage. The absence of computed tomography, the gold standard for visceral adipose tissue (VAT) and subcutaneous adipose tissue (SAT) measurements, restricted the precision of adipose tissue assessment. Biopsies of VAT and SAT were not available, limiting direct tissue analysis. Additionally, glycated hemoglobin measurements were not performed, precluding further evaluation of long-term glycemic control.

## Data Availability

The original contributions present in this study are included in the article.

## Supplementary Materials

The following supporting information can be downloaded at https://www.mdpi.com/article/10.3390/metabo15110695/s1: Figure S1: Recruitment flowchart diagram describing the inclusion of participants; Figure S2: Clinical variables considered in the present study, Figure S3: Metabolite analysis of control and case groups, Table S1: Multivariate logistic regression of metabolic predictors of weight gain.

## Abbreviations

The following abbreviations are used in this manuscript:

ART	Antiretroviral therapy
BCAA	Branched-chain amino acid
BIC/TAF/FTC	Bictegravir/tenofovir alafenamide/emtricitabine
DHA	Docosahexanoic acid
DTG/ABC/3TC	Dolutegravir/abacavir/lamivudine
GGT	Gamma-glutamyl transpeptidase
INSTI	Integrase strand transfer inhibitors
HBV	Hepatitis B Virus
HCV	Hepatitis C Virus
HEV	Hepatitis E Virus
HIV	Human Immunodeficiency Virus
HMDB	Human Metabolome Database
HOMA-IR	Homeostasis Model Assessment for Insulin Resistance
LC-MS/MS	Liquid chromatography–mass spectrometry
OR	Odds ratio
PCA	Principal Component Analysis
PWH	People living with HIV
RORA	Retinoic Acid-related Orphan Receptor alpha
SAT	Subcutaneous adipocyte tissue
T2DM	Type 2 diabetes mellitus
TC	Total cholesterol
TGP or ALT	Alanine aminotransferase
TGO or AST	Aspartate aminotransferase
TG	Triglycerides
USG	Ultrasonography
VAT	Visceral adipose tissue
VL	Viral load
