# Supplementary material for: Metabolomic Profile of Weight Gain of People Living with HIV Treated with Integrase Strand Transfer Inhibitor Regimens Reveals Dysregulated Lipid Metabolism and Mitochondrial Dysfunction
Source: Metabolites. 2025 Oct 25;15(11):695. doi: 10.3390/metabo15110695 (PMC12654600; doi:10.3390/metabo15110695)
Supplement: Supplementary file 1 [file metabolites-15-00695-s001.zip › metabolites-3901669-supplementary.pdf]

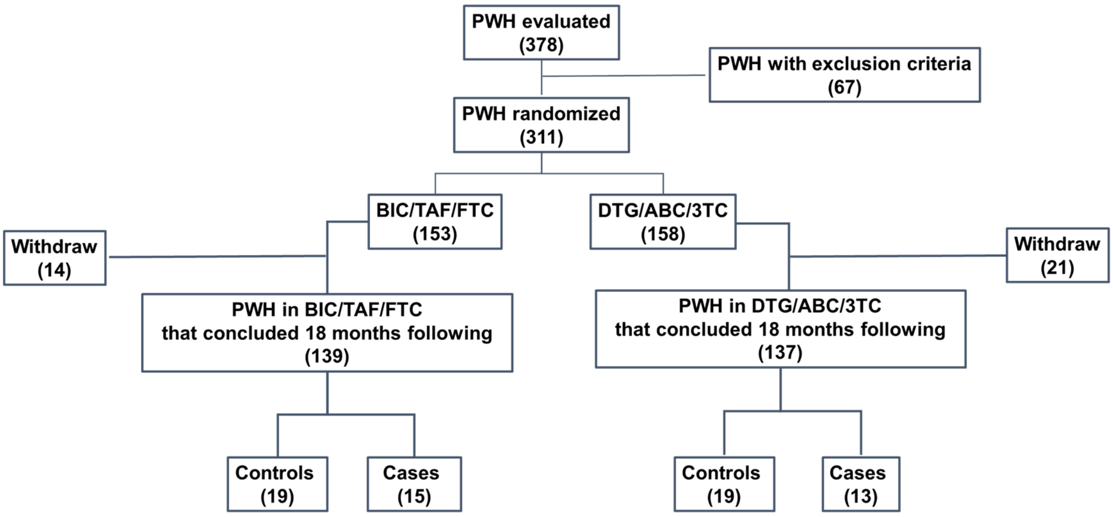

Figure S1. Recruitment flowchart diagram describing the inclusion of participants. Abbreviations: BIC/TAF/FTC, bictegravir/ alafenamide tenofovir/emtricitabine; DTG/ABC/3TC, dolutegravir/abacavir/ lamivudine; PWH, people living with HIV.

Table Supplementary S1. Multivariate logistic regression of metabolic predictors of weight gain.

Characteristics	aOR (95% CI)	*P*
HOMA-IR > 2.6	2.69 (0.756 - 9.63)	0.126
VAT ≥ 4 cm	4.82 (1.10 – 20.95)	0.036
TG ≥ 150 mg/dL	4.24 (1.22 - 14.76)	0.023
Baseline HIV RNA viral load ≥ 100,000 copies/mL	4.82 (1.10 - 20.95)	0.036

Abbreviations: OR: Odds Ratio; CI: Confidence interval; HOMA-IR: Homeostatic Model Assessment for Insulin Resistance, VAT: visceral adipose tissue; VL: Viral Load, TG: triglycerides.

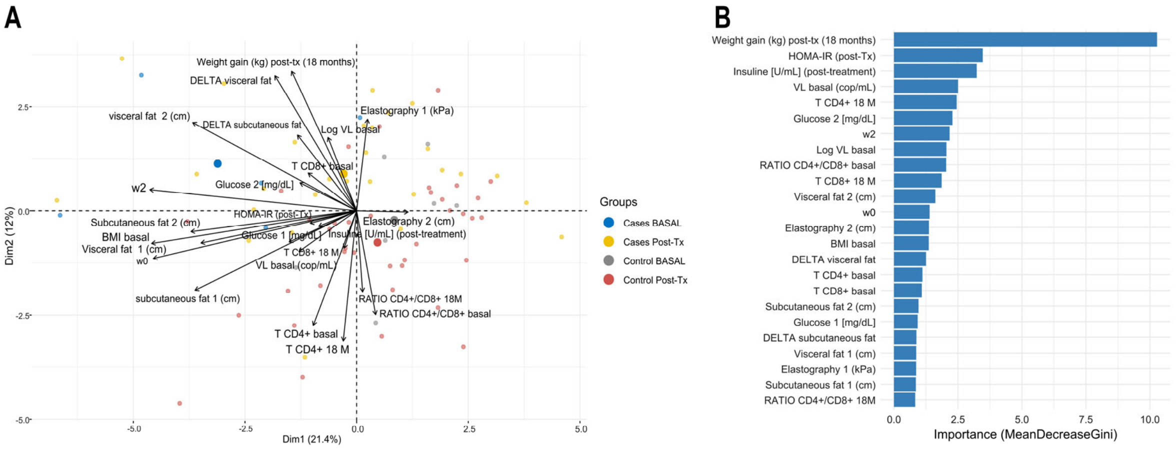

Supplementary Figure S2. Clinical variables considered in the present study. A. Biplot of Principal Component Analysis (PCA). PCA biplot showing post-treatment cases (yellow) clustering in the upper-left

region, associated with weight gain, increased visceral/subcutaneous fat (Δ), and higher HOMA-IR, while post-treatment controls (red) group to the right with more stable metabolic parameters. B. Random Forest variable importance for classifying cases and controls, showing post-treatment weight gain at 18 months as the strongest predictor, followed by HOMA-IR and post-treatment insulin.

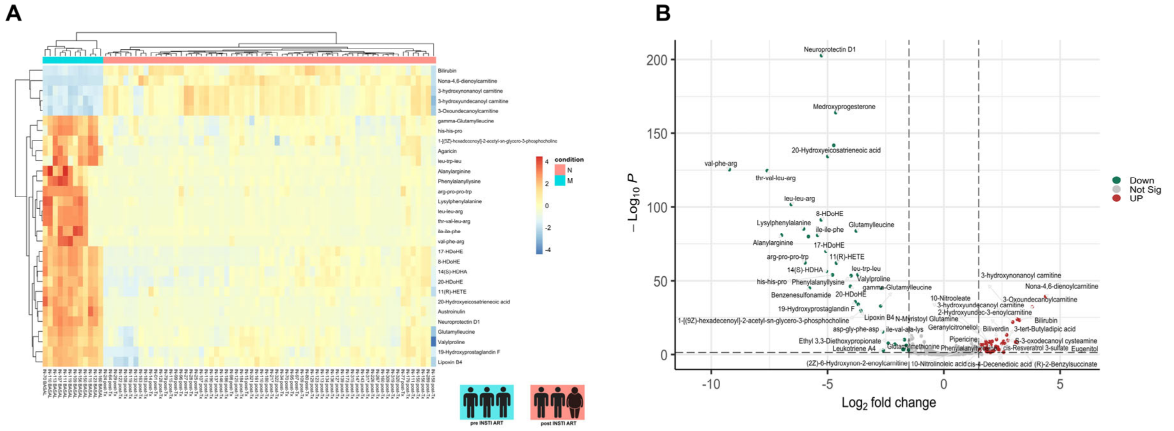

Supplementary Figure S3. Metabolite analysis of control and case groups. A. Heatmap of metabolomic profiles with hierarchical clustering. Columns represent individuals: cases (M) and controls (N). Distinct patterns are observed in metabolites related to lipid metabolism (carnitines, phosphatidylcholines, and eicosanoids), showing a tendency to differentiate individuals with weight gain from controls. Colors represent normalized relative abundance (Z-score), with red indicating higher and blue lower levels. B. Volcano plot of differential metabolites between cases and controls. A significant increase in acylcarnitines in cases, whereas metabolites related to anti-inflammatory and pro-resolving pathways (e.g., lipoxins, neuroprotectins, eicosanoid derivatives) appeared reduced.
